# Primary Cutaneous Rhabdoid Squamous Cell Carcinoma: A Case Report and Review of Molecular Features

**DOI:** 10.7759/cureus.73592

**Published:** 2024-11-13

**Authors:** Svetlana Bobkova, Eli P Oldham, Carlos Gomez-Meade, Igor Shendrik

**Affiliations:** 1 Biomedical Sciences, Oklahoma State University Center for Health Sciences, Tulsa, USA; 2 Medical Student Research, Oklahoma State University Center for Health Sciences, Tulsa, USA; 3 Mohs Surgery, Oklahoma Cancer Specialists Skin Cancer Center, Broken Arrow, USA; 4 Dermatopathology, Regional Medical Laboratory and Pathology Laboratory Associates, Tulsa, USA

**Keywords:** immunohistochemistry, molecular transformation, primary cutaneous tumors, rhabdoid, squamous cell carcinoma

## Abstract

We present a rare case of rhabdoid squamous cell carcinoma (RSCC) on the scalp of a non-immunosuppressed male patient in his late 60s. This aggressive variant of squamous cell carcinoma (SCC) is characterized by tumor cells with eccentrically located nuclei and abundant eosinophilic cytoplasm, as observed on histopathological examination. While rhabdoid morphology has been reported in various anatomical sites, its occurrence in primary cutaneous tumors is exceptionally uncommon, with fewer than 10 cases documented to date. Immunohistochemically, the neoplasm demonstrated positivity for p40, cytokeratin 5/6 (CK5/6), and vimentin, while retaining integrase interactor 1 (INI1) expression and showing negativity for muscle differentiation markers. Loss of p53 staining suggested its role in the progression to a more aggressive phenotype. The DecisionDx-SCC assay (Castle Biosciences, Friendswood, TX, US) classified the tumor as Class 2B, indicating the highest biological risk for metastasis and poor outcomes. Despite the limited number of reported cutaneous RSCC cases, this variant appears to exhibit high aggressiveness, mirroring other visceral tumors with rhabdoid features. We discuss potential molecular events underlying this transformation. This case highlights the importance of recognizing this rare variant and its implications for patient management and prognosis.

## Introduction

Squamous cell carcinoma (SCC) is one of the most common malignancies of the skin, typically arising in sun-exposed areas and generally associated with a favorable prognosis [1]. However, certain variants, such as rhabdoid SCC (RSCC), exhibit more aggressive behavior and pose significant challenges in diagnosis and management [2]. RSCC is characterized by rhabdoid cells with eccentric nuclei and abundant eosinophilic cytoplasm, requiring immunohistochemical evaluation for accurate diagnosis [2]. Although documented in various malignancies, primary cutaneous SCC with rhabdoid morphology is exceedingly rare, with fewer than 10 cases reported [2].

RSCC has been identified in various anatomical sites, including the oral cavity and genitourinary tract, but its occurrence on the skin, particularly in the absence of immunosuppression, is infrequently reported [3]. In this case report, we present a rare case of RSCC on the scalp of an elderly male patient without a history of immunosuppression. The tumor was evaluated using histopathological and immunohistochemical techniques, along with a 40-gene expression DecisionDx-SCC assay (Castle Biosciences, Friendswood, TX, US) to assess the biological risk for metastasis [4].

## Case presentation

**Figure 1:**
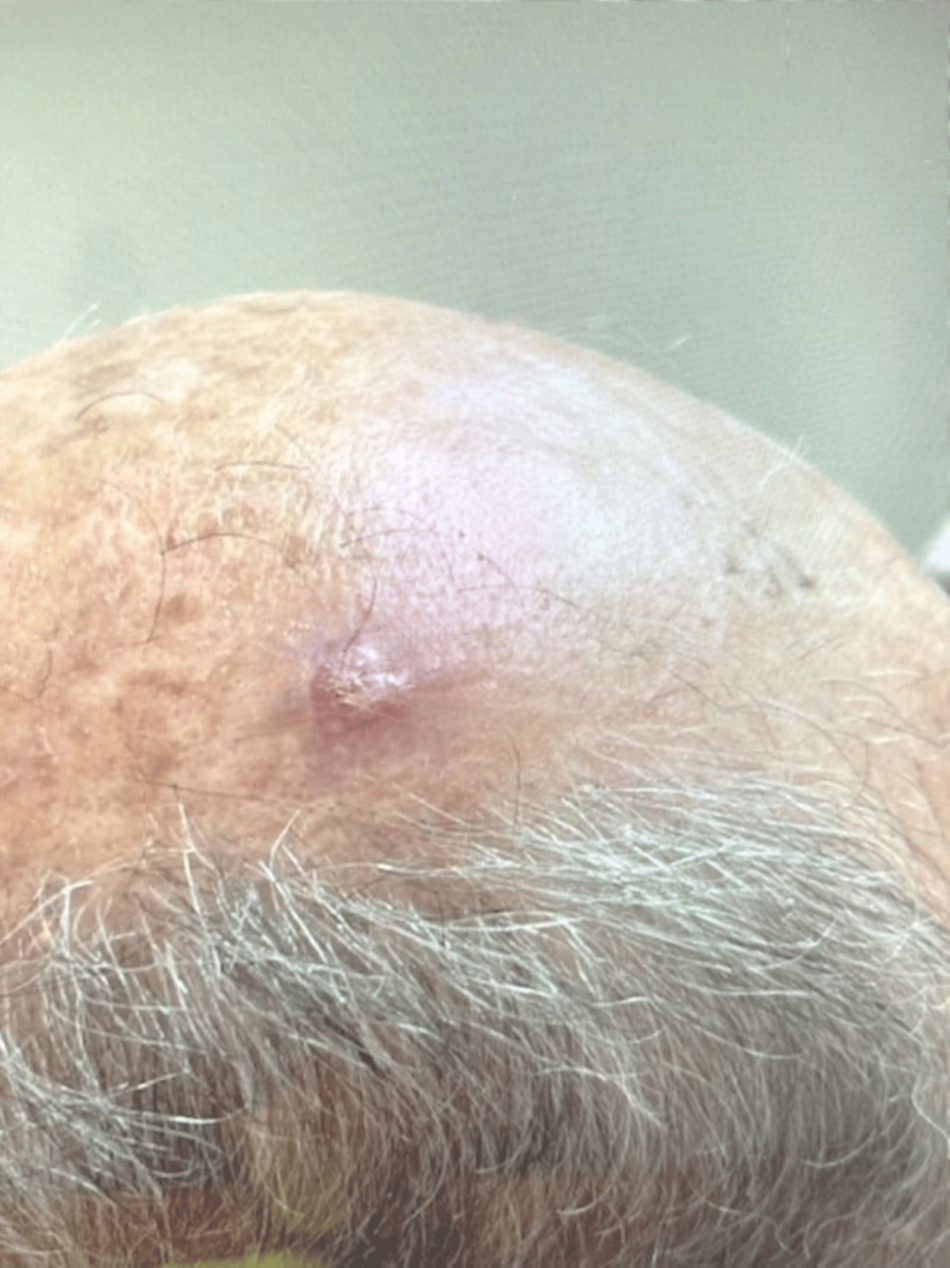
A raised pink-to-purple nodule with central crusting on the patient's scalp, with surrounding erythema and slight swelling.

The patient, a man in his late 60s, presented with a raised pink-to-purple nodule on his scalp with central crusting (Figure 1).

**Figure 2:**
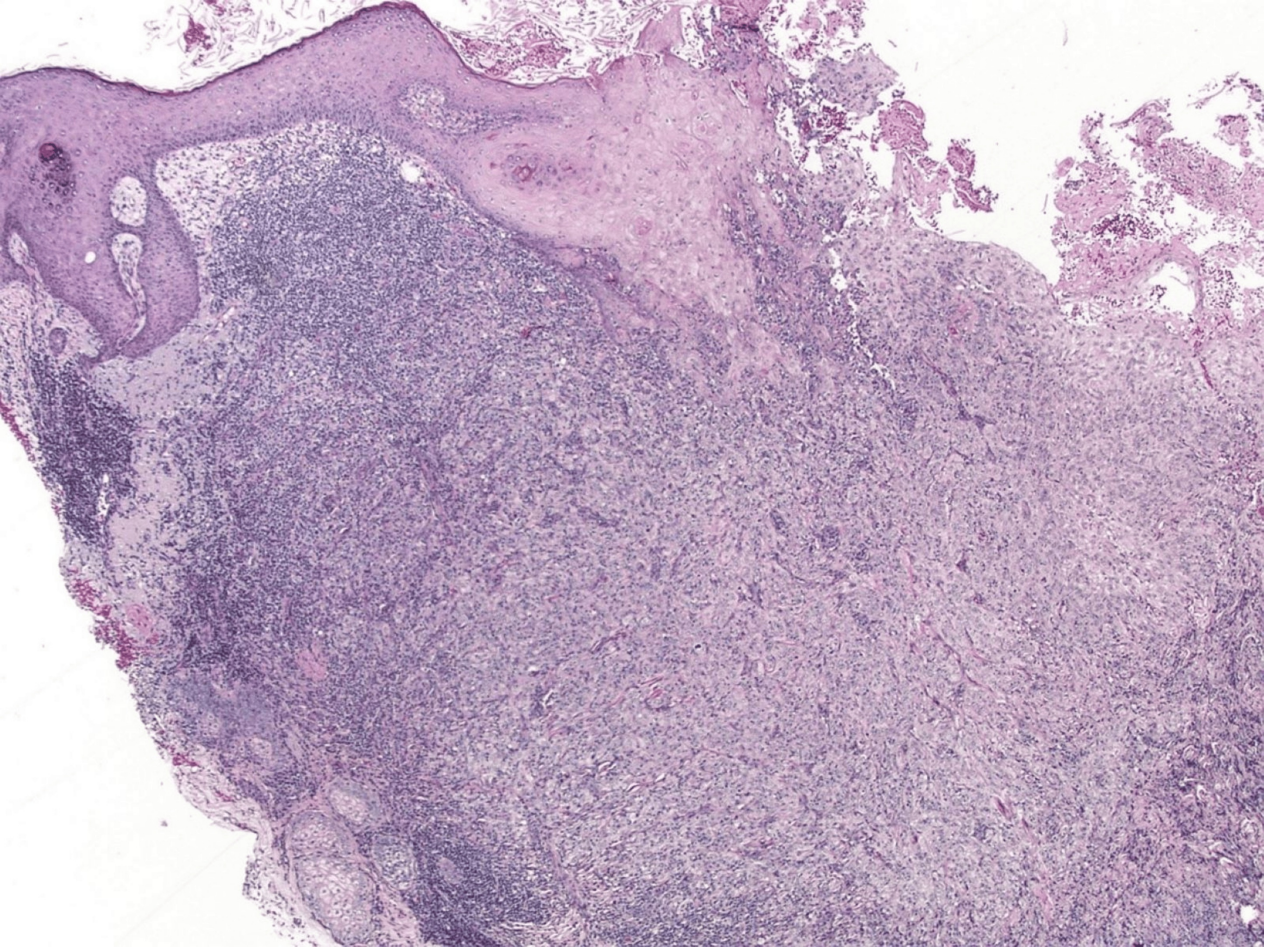
: Ulcerated actinically damaged skin with tumor invasion into the reticular dermis, surrounded by chronic inflammation (x20).

The initial biopsy demonstrated SCC in situ with underlying areas of invasion, interpreted as moderately differentiated invasive SCC. Mohs excision was performed based on this initial diagnosis, demonstrating ulcerated, actinically damaged skin with an ill-defined tumor measuring approximately 2.2 cm at the largest diameter and invading the reticular dermis (Figure 2).

**Figure 3:**
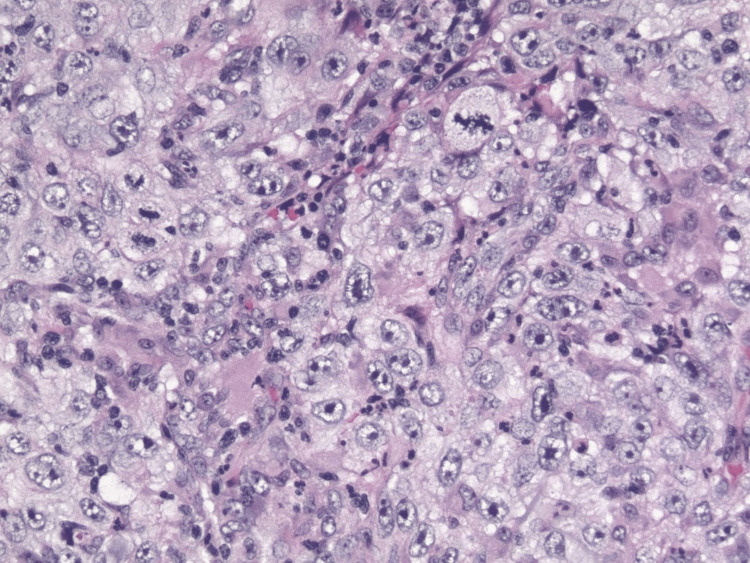
Malignant cells exhibiting rhabdoid cytologic appearance (x200).

Malignant cells displayed a rhabdoid cytologic appearance, characterized by large cells with eccentric nuclei and eosinophilic cytoplasmic inclusions (Figure 3).

**Figure 4:**
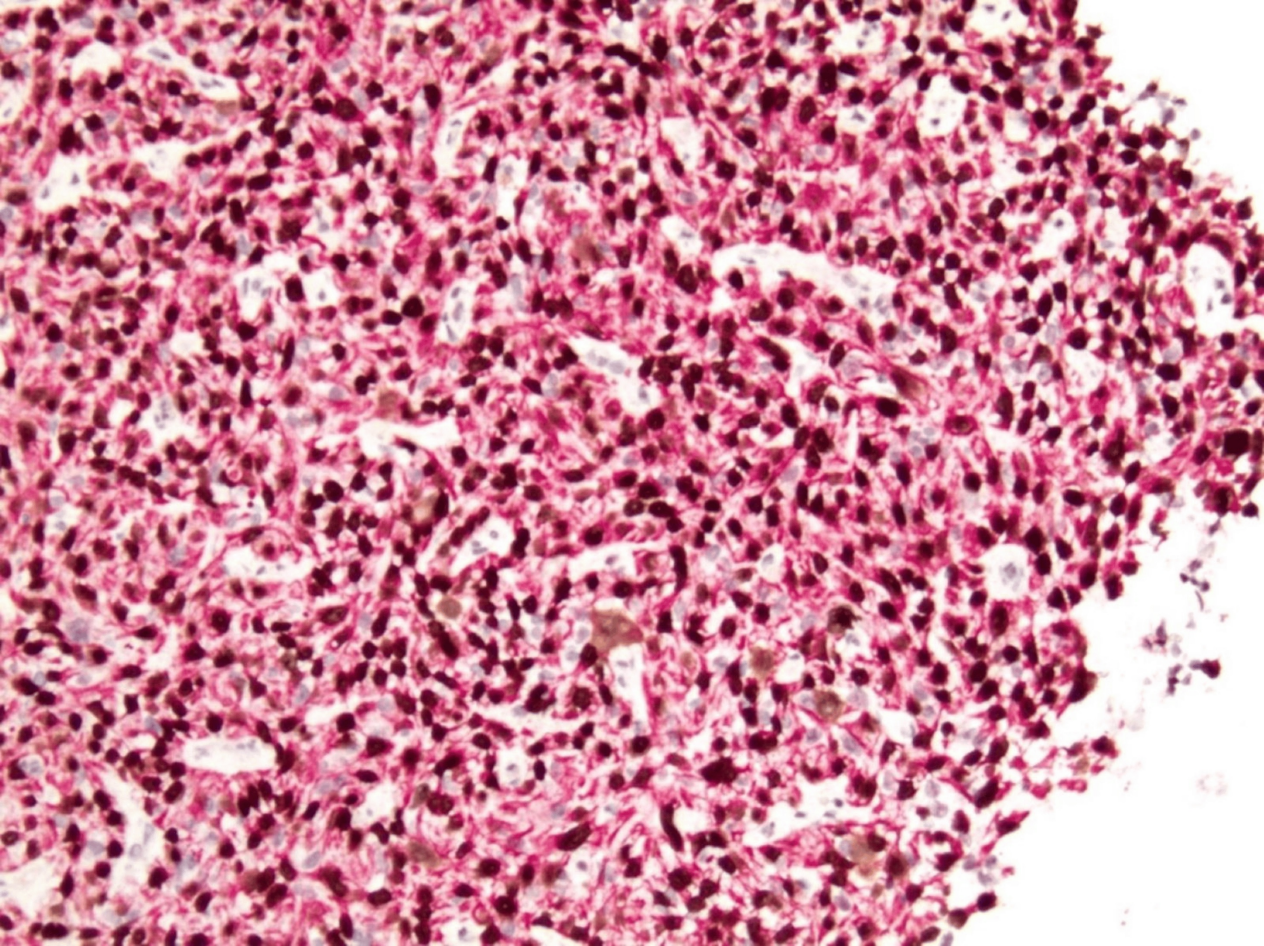
Immunohistochemical stain showing uniform positivity for p40 (dark brown nuclear stain) and cytokeratin 5/6 (red cytoplasmic stain) in the malignant cells (x100).

**Figure 5:**
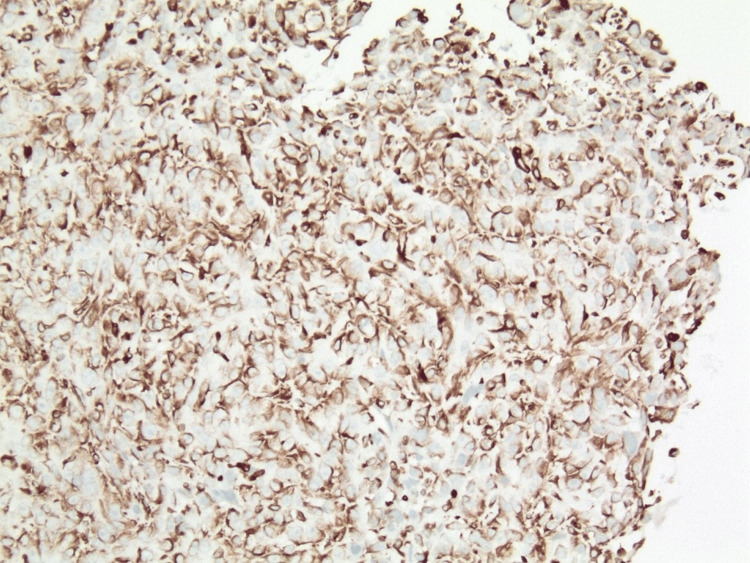
Immunohistochemical stain for vimentin of the same area as in Figure 4 (x100).

Immunohistochemical stains revealed positivity for p40, cytokeratin 5/6 (CK5/6) (Figure 4), and vimentin (Figure 5), with retained integrase interactor 1 (INI1) expression and membranous expression of beta-catenin and E-cadherin. No expression of SALL4, OCT3/4, ERG, CD117, CD34, MYF-4, Desmin, Calponin, SMA, CD68, or p53 was seen.

Following Mohs excision, the central portion of the tumor (debulk specimen) was sent for the 40-gene expression DecisionDx-SCC assay, which categorized the tumor as Class 2B, indicating the highest biological risk for metastasis and poor outcomes [4]. Given this high-risk classification, adjuvant radiotherapy was administered to minimize recurrence risk. To date, no tumor recurrence or lymph node metastases have been noted, although the follow-up period is relatively short at two months.

## Discussion

Only a few cases of cutaneous RSCC have been reported in the literature, and these cases usually indicate aggressive tumor behavior [2]. Histologically, cutaneous RSCC is characterized by large cytoplasmic eosinophilic hyaline inclusions with peripheral displacement of vesicular nuclei [3]. Immunohistochemically, these tumor cells show diffuse reactivity for cytokeratin and vimentin localized to the cytoplasmic inclusions, but not for desmin, smooth muscle actin, or other skeletal muscle markers [5]. The differential diagnosis of cutaneous rhabdoid neoplasms includes a variety of primary cutaneous and metastatic lesions, such as primary cutaneous malignant rhabdoid tumor, epithelioid sarcoma, rhabdoid melanoma, and others [5]. In our case, the tumor cells demonstrated positivity for p40, CK5/6, and cytoplasmic vimentin expression with membranous accentuation but lacked muscle-specific markers. This immunohistochemical profile, combined with the tumor occurrence on a background of SCC in situ diagnosed by the initial biopsy, confirms the diagnosis of primary cutaneous RSCC.

Although some cases of RSCC have been reported in transplant patients, suggesting a potential role for immunosuppression [1], our patient was not immunosuppressed. Unlike primary rhabdoid tumors, such as malignant rhabdoid tumors, atypical teratoid/rhabdoid tumors, SMARCA4-deficient thoracic sarcomas, and epithelioid sarcoma, which often show alterations in SMARCB1 (INI1) or SMARCA4, cutaneous RSCC does not exhibit such inactivation [6,7]. In our case, the preservation of INI1 expression supports this finding.

Cytoplasmic vimentin positivity suggests that the tumor has acquired some mesenchymal traits, indicative of epithelial-mesenchymal transition (EMT) [8]. However, EMT also requires the loss of epithelial markers, such as E-cadherin, and the activation of pathways like Wnt/beta-catenin. In our case, the tumor cells exhibited preserved membranous beta-catenin and E-cadherin expression, with no evidence of nuclear translocation or abnormal cytoplasmic accumulation, indicating that the Wnt/beta-catenin pathway is inactive, consistent with partial but not complete EMT [8].

The absence of p53 is significant, as it leads to genomic instability, facilitating oncogenic mutations and contributing to dedifferentiation and tumor aggressiveness [7]. While p53 loss is not uncommon, it creates a permissive environment for further mutations that drive the aggressive phenotype and unusual morphology observed in this case. The preserved Wnt pathway suggests that mechanisms other than complete EMT are contributing to the tumor's behavior.

The high-risk classification (Class 2B) by the DecisionDx-SCC assay, indicating the highest biological risk for metastasis (approximating 50% over a three-year period), emphasizes the importance of aggressive treatment measures, including adjuvant radiotherapy, and the need for vigilant follow-up [4]. Given the limited activation of pathways like Wnt and the presence of partial EMT-like changes, further research is needed to understand the molecular mechanisms driving this phenotype and to develop optimal management strategies.

## Conclusions

We describe a rare case of primary cutaneous SCC, characterized by the rhabdoid appearance of the invasive tumor indicative of dedifferentiation and partial EMT traits. Pertinent clinical, histologic, and immunohistochemical features, including vimentin positivity and p53 loss, are discussed. The use of DecisionDx-SCC assay provided crucial prognostic information, classifying the tumor as Class 2B, indicating the highest biological risk for metastasis and poor outcomes. Recognizing this variant is important due to its aggressive clinical behavior and poor prognosis, which necessitates a tailored management approach involving aggressive treatment and vigilant follow-up, along with further research into the underlying molecular mechanisms to develop targeted therapies.
